# Cross-Talk of Low-Level Sensory and High-Level Cognitive Processing: Development, Mechanisms, and Relevance for Cross-Modal Abilities of the Brain

**DOI:** 10.3389/fnbot.2020.00007

**Published:** 2020-02-14

**Authors:** Xiaxia Xu, Ileana L. Hanganu-Opatz, Malte Bieler

**Affiliations:** ^1^Developmental Neurophysiology, Center for Molecular Neurobiology, University Medical Center Hamburg-Eppendorf, Hamburg, Germany; ^2^Laboratory for Neural Computation, Institute of Basic Medical Sciences, University of Oslo, Oslo, Norway

**Keywords:** cross-modal processing, primary sensory cortices, prefrontal cortex, top-down, bottom-up, development

## Abstract

The emergence of cross-modal learning capabilities requires the interaction of neural areas accounting for sensory and cognitive processing. Convergence of multiple sensory inputs is observed in low-level sensory cortices including primary somatosensory (S1), visual (V1), and auditory cortex (A1), as well as in high-level areas such as prefrontal cortex (PFC). Evidence shows that local neural activity and functional connectivity between sensory cortices participate in cross-modal processing. However, little is known about the functional interplay between neural areas underlying sensory and cognitive processing required for cross-modal learning capabilities across life. Here we review our current knowledge on the interdependence of low- and high-level cortices for the emergence of cross-modal processing in rodents. First, we summarize the mechanisms underlying the integration of multiple senses and how cross-modal processing in primary sensory cortices might be modified by top-down modulation of the PFC. Second, we examine the critical factors and developmental mechanisms that account for the interaction between neuronal networks involved in sensory and cognitive processing. Finally, we discuss the applicability and relevance of cross-modal processing for brain-inspired intelligent robotics. An in-depth understanding of the factors and mechanisms controlling cross-modal processing might inspire the refinement of robotic systems by better mimicking neural computations.

## Sensory-Cognitive Interplay During Cross-Modal Processing

**FIGURE 1:**
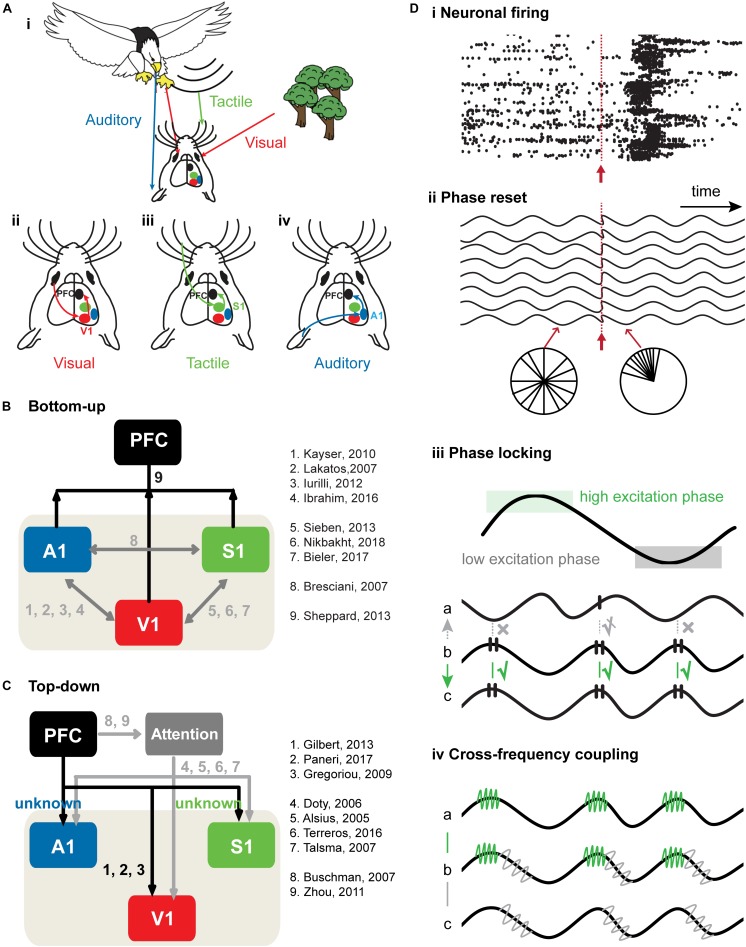
Bottom-up and top-down cross-modal processing. **(A)** Schematic drawing of a mouse receiving visual information (red arrow) about a behaviorally-irrelevant object (trees) and a behaviorally relevant object (approaching eagle) that is accompanied by tactile and auditory information (vibrations and sounds, green and blue arrows). (i) Schematic diagram showing how visual (ii), tactile (iii), and auditory (iv) information is transferred in the brain. **(B)** Schematic diagram of bottom-up sensory information flow from primary sensory cortices to PFC. The black arrows correspond to cross-modal processing from primary sensory cortices to PFC, whereas the gray arrows correspond to cross-modal processing within primary sensory cortices. **(C)** Schematic diagram of top-down prefrontal modulation of neuronal activity in primary sensory cortices. PFC has been proposed as the source of top-down attention signals that modulate cross-modal processing in primary sensory cortices in favor of the attended features. Studies have highlighted the effects of attention on neuronal responses in primary sensory cortices, such as an increase in neuronal discharges and a decrease in the variability of neuronal responses. The black arrows correspond to the direct connections from PFC to primary sensory cortices. The gray arrows correspond to the top-down modulation of sensory processing in primary sensory cortices during attention. **(D)** Neural mechanisms of bottom-up and top-down cross-modal processing. (i) Spike trains before and after stimulus. Neuronal firing is random pre-stimulus, whereas post-stimulus firing rate is enhanced and marked by a precisely timed onset. Stimulus is represented by the red arrow. (ii) Phase reset as a mechanism of bottom-up cross-modal processing. The phase of oscillatory activity is random pre-stimulus, but resets post-stimulus. Stimulus is represented by the red arrow. (iii) Phase locking as a mechanism of bottom-up and top-down sensory processing. Black lines on the peak of the ongoing oscillation indicate spikes. Effective communication occurs when spiking activity of area b arrives at the high excitatory phase of area c and induces spikes in area c. Ineffective communication occurs when spiking activity in area b arrives at the low excitation phase of the signal a and fails to induce spikes in area a. When spiking activity in area b arrives at the rising phase of area a, communication between effective and ineffective levels (indicated by crossed-out check mark) occurs. (iv) Communication between two areas using cross-frequency coupling (CFC). Signal b shows that green and gray high frequency rhythms “ride” on the black low frequency rhythm. CFC between signal a and b enables area a and b to communicate through high frequency rhythm (marked in green). CFC between signal b and c enables b and c to communicate through high frequency rhythm.

The brain permanently receives sensory information addressing multiple modalities. Its capability to process diverse sensory inputs is mandatory to create a coherent perception of the environment, and ultimately to guide adaptive behavior. The diverse sensory components of a stimulus are processed and conveyed in a discrete manner by modality-specific pathways (Figure 1A), where each modality provides unique information about the stimulus. Complementing stimulus information reduces stimulus uncertainty and enhances behavioral responses, thus leading to faster and more accurate decision-making (Stein et al., 1988; Gleiss and Kayser, 2012; Siemann et al., 2014; Hammond-Kenny et al., 2017; Meijer et al., 2018). The process of sensory convergence, where inputs of different senses are combined without being able to easily dismantle them into independent unimodal components, is termed as *cross-modal integration* (Keil and Senkowski, 2018; Nikbakht et al., 2018). In order to evoke a coherent cross-modal perception, neural areas accounting for sensory and cognitive processing need to optimally interact with each other. This appears to be a challenging computation given the multidimensionality of neural activity and the fact that neural areas specialized in processing one component of a stimulus are located at distant parts in the brain (Harris and Mrsic-Flogel, 2013; Runyan et al., 2017; Stringer et al., 2019). In addition, the neural interactions of systems accounting for sensory and cognitive processing are highly dynamic, emerging at early age and developing over time (Goodman and Shatz, 1993; Siegel et al., 2012; Parisi et al., 2019). Comparable sensory systems and the ease of measuring behavioral effects motivated the use of large mammalian species as prime models to study the mechanisms of cross-modal processing and their emergence during development (Stein et al., 1993; Wallace and Stein, 1997; Calvert and Thesen, 2004). Here we focus on the interdependence of primary sensory cortices (S1, V1, A1) and PFC in rodents, aiming to critically review our current understanding of the mechanisms that enable the communication between remote brain areas dedicated to sensory and cognitive processing during cross-modal perception. In addition, we will review how bottom-up and top-down mechanisms underlying cross-modal processing emerge during development. Despite possible differences of neuronal processing when compared to larger mammals such as cats or monkeys, the use of rodent models bears several advantages for the study of cross-modal processing. Recent developments in rodent behavior and genetics, viral methods, and genetically encoded Ca^2+^ indicators offer the possibility to study causal relations in the brain, monitor neuronal activity over time, and explore the relationship between neural network properties and behavior underlying cross-modal processing (Fenno et al., 2011; Chen et al., 2013). Relying on these state-of-the-art methods, our understanding of the cellular and network mechanisms underlying cross-modal processing as well as their development should be fostered. Detailed insights on the neural computations are critical for the development of autonomous agents and their optimal interaction with the environment under conditions of sensory uncertainty. Thus, by providing knowledge of neuronal computations underlying cross-modal integration, this review aims to uncover general principles of neuronal processing and to inspire multidisciplinary research in the field of robotics.

### Bottom-Up Cross-Modal Processing in Primary Sensory Cortices

Sensory interactions have primarily been demonstrated in high-level association cortices, such as PFC or posterior parietal cortex (PPC) (Lippert et al., 2013; Yau et al., 2015; Song et al., 2017). However, cross-modal processing has been shown to take place already at early stages of sensory processing, such as in the brainstem (Aitkin et al., 1981; Jain and Shore, 2006; Koehler et al., 2011), thalamus (Komura et al., 2005; Allen et al., 2017; Bieler et al., 2018) or primary sensory cortices (Lakatos et al., 2007; Kayser et al., 2010; Sieben et al., 2013).

The superior colliculus (SC) of the midbrain received particular attention when investigating the principles of multisensory processing. The SC receives multiple ascending (Edwards et al., 1979; Mize, 1983) and descending (Clemo and Stein, 1984; Meredith and Clemo, 1989) unisensory afferent sources that converge onto individual neurons, making the SC a prime model to study mechanisms of cross-modal processing. Deep-layer multisensory neurons of the SC control sensory as well as motor responses. Cross-modal but not unimodal, or multiple unimodal stimuli of the same modality (Alvarado et al., 2007), cause an enhancement of neuronal firing (Meredith and Stein, 1983; Perrault et al., 2005), which consequently mediates orienting behavior (Stein et al., 1988; Gingras et al., 2009). It has been shown that the inputs from cortical association areas are critical to manifest cross-modal responses in the SC (Stein et al., 2002; Alvarado et al., 2009). While SC neurons in behaving animals continue to respond to multiple sensory modalities following cortical inactivation, multisensory responses are suppressed, and multisensory integration is eliminated (Jiang et al., 2002, 2007).

The described neuronal responses to cross-modal stimuli in first-order thalamic nuclei and primary sensory cortices occur at too short latency to result from processing feedback information. Thus, also low-level putatively unimodal brain areas integrate cross-modal information in a bottom-up manner. The bottom-up detection and discrimination of stimuli are fundamental stages of sensory processing, because they allow, on the one hand, for rapid detection of a stimulus, and on the other hand, for discrimination between similar stimuli based on fine details (Guo et al., 2017). The detection and discrimination of a stimulus are improved when it provides features from multiple modalities (Gleiss and Kayser, 2012; Sheppard et al., 2013; Siemann et al., 2014; Hollensteiner et al., 2015; Nikbakht et al., 2018).

Similar mechanisms of cross-modal processing first described in the cat SC have also been found in rodent SC (Gharaei et al., 2018) as well as in primary sensory cortices, thus challenging the strict hierarchical model of sensory processing (Foxe and Schroeder, 2005). For example, co-presentation of an auditory stimulus enhances orientation selectivity of V1 neurons (Ibrahim et al., 2016). This cross-modal enhancement of neuronal firing was strongest under low-contrast conditions, suggesting that cross-modal information is particularly beneficial for perceptually-guided behavior under ambiguous situations. In addition to cue-integration, cross-modal processing also depends on modality segregation, i.e. the suppression of neuronal activity in one modality-specific primary sensory cortex due to the concurrent presentation of a stimulus of a non-matching sensory modality (Iurilli et al., 2012; Song et al., 2017; Bieler et al., 2018; Gharaei et al., 2018). For example, Gharaei et al. (2018) demonstrated that unisensory stimulation enhances neuronal responses in the SC, whereas cross-modal stimulation rarely enhances but rather suppresses neuronal firing discharges. At the level of primary sensory cortex, Iurilli et al. (2012) showed that evoked activity in A1 enhances local inhibitory firing in deep layers of V1, which in turn decreases the activity of V1 supragranular pyramidal neurons. Consequently, at behavioral level, visually-conditioned responses were suppressed by acoustic stimulation. Experimental research examining the mechanisms of sensory convergence in low-level sensory regions emphasized the processing and relay of basic object feature information (Iurilli et al., 2012; Sieben et al., 2013; Bieler et al., 2018; Morrill and Hasenstaub, 2018). However, the formation, storage, and utilization of cross-modal object representations during behavior require an interaction of neuronal areas accounting for sensory and cognitive processing (Hindley et al., 2014; Reid et al., 2014; Jacklin et al., 2016). Thus, while both sensory integration and separation are part of bottom-up cross-modal processing in primary sensory cortices, the mechanisms underlying the functional communication between low- and high-level brain areas during cross-modal perception are still largely unknown.

### Top-Down Modulation of Cross-Modal Processing in Primary Sensory Cortices

Creating a consistent mental representation of the multisensory environment depends on more than the convergence of sensory information in primary sensory cortices (Choi et al., 2018). Sensory processing in primary sensory cortices is modulated top-down to create a multisensory perception, and finally, behavioral action (Ernst and Newell, 2007; Gilbert and Li, 2013; Talsma, 2015; Bizley et al., 2016; Kunicki et al., 2019). In particular, top-down influences from high- to low-level brain areas allow for the preferential processing, and thereby the facilitation of specific sensory inputs in primary sensory cortices (Talsma et al., 2010). Such top-down information may be related to attention, expectation or perceptual demands (Paneri and Gregoriou, 2017; Choi et al., 2018). Attention is a core property of all perceptual and cognitive operations. Given the limited capacity to process competing environmental inputs, attentional mechanisms allow for the selection and modulation as well as for sustained focus on information most relevant for behavior (Chun et al., 2011). Attention modulates neuronal activity and improves the signal-to-noise ratio thereby increasing signal efficacy for attended stimuli and enhancing the representation of attended features (Noudoost et al., 2010). Attention facilitates the integration of multisensory inputs in a top-down manner (Fiebelkorn et al., 2010; Mühlberg and Soto-Faraco, 2019). Top-down modulation enables the flexible selection of information based on task goals, as well as providing an order for selectively modulating multiple stimuli within each modality if they are competing for processing resources (Alsius et al., 2005; Doty et al., 2006). For example, Terreros et al. (2016) showed that mice are able to selectively focus on a visual stimulus, ignoring distractive auditory stimuli during selective attention in a two-choice visual discrimination task (Terreros et al., 2016). Furthermore, top-down modulation reweights sensory information and facilitates the integration of cross-modal inputs (Alsius et al., 2005; Busse et al., 2005; Bresciani and Ernst, 2007; Talsma et al., 2007; Lakatos et al., 2009; Fiebelkorn et al., 2010; Muhlberg et al., 2014). Prior cross-modal exploration of task-relevant objects significantly facilitates the detection performance of a rat in a cross-modal object recognition task (Jacklin et al., 2016). Moreover, rats are able to recognize a visually presented object, which has been only explored by the tactile sense (Winters and Reid, 2010). Top-down task demands further modulate cross-modal processing in primary sensory cortices. For example, during the free exploration of novel objects in the dark (whisker-based tasks), V1 and S1 responses carried comparable amounts of information about object identity (Vasconcelos et al., 2011). However, during the execution of an aperture tactile discrimination task, which is based on top-down task demands, S1 showed faster and more robust tactile recruitment when compared to V1.

Several frontal and parietal cortical regions, such as PPC and PFC, have been proposed as the source of top-down modulatory signals (Noudoost et al., 2010; Winters and Reid, 2010; Jacklin et al., 2016; Paneri and Gregoriou, 2017; Mohan et al., 2018). For example, it has been shown that top-down modulation originating in PPC influences cross-modal processing in primary sensory cortices (Mohan et al., 2017; Kunicki et al., 2019), and damage to PPC leads to performance deficits in sensory discrimination tasks (Binkofski et al., 2001; Winters and Reid, 2010). Given the well-established role of PFC in cognitive control and executive function (Miller and Cohen, 2001), it has been hypothesized that it modulates sensory processing in primary sensory cortices as well (Buschman and Miller, 2007). Bichot et al. (2015) showed in non-human primates performing a visual search task, that feature-based attention adjusts the neural firing activity of prefrontal neurons representing an attended feature to quickly locate a target object (Bichot et al., 2015). Moreover, neural responses in PFC emerge earlier when compared to the responses in visual cortex during covert attention tasks (Gregoriou et al., 2009; Monosov et al., 2010; Zhou and Desimone, 2011; Lennert and Martinez-Trujillo, 2013; Bichot et al., 2015; Siegel et al., 2015). Pharmacological inactivation of PFC induced space-specific impairments in a covert visual search task, and was particularly prominent when a shift in attention was required (Monosov and Thompson, 2009). The PFC might provide top-down modulatory signals to primary sensory cortices through direct axonal projections. For example, Zhang et al. (2014) showed that activation of prefrontal local GABAergic circuits powerfully influences sensory processing in V1 through direct connectivity from PFC to V1 (Zhang et al., 2014). Moreover, prefrontal modulatory signals may reach primary sensory cortices via the sensory thalamus. Stimulating the PFC has been shown to increase tactile responses and alter basal activity in the ventrobasal region of the thalamus (Cao et al., 2008). In line with this, optogenetic manipulation of prefrontal activity perturbs the ability of mice to appropriately select between conflicting visual and auditory stimuli during a cross-modal divided-attention task that is known to depend on prefrontal-thalamic interactions (Wimmer et al., 2015).

### Anatomical Substrate of Interactions Between Neuronal Networks Accounting for Sensory and Cognitive Processing

Direct bottom- up (Henschke et al., 2015; Mowery et al., 2016; Bieler et al., 2017b; Henschke et al., 2017) and top-down cortico-cortical (Zhang et al., 2014; Makino and Komiyama, 2015) as well as indirect cortico-thalamo-cortical pathways (Theyel et al., 2010; Roth et al., 2016) represent the anatomical substrate of the functional communication between low- and high-level brain areas during cross-modal processing (Figures 1B,C).

Short latency cross-modal interactions in low-level sensory cortices rely on direct long-range connections (Sieben et al., 2013; Stehberg et al., 2014; Henschke et al., 2015). For example, visual stimulation modulates S1 activity via direct cortico-cortical connections, while pharmacological inactivation of V1 diminishes cross-modal effects in S1 (Sieben et al., 2013). In addition, optogenetic stimulation of A1-V1 projection neurons sharpens the orientation selectivity of neurons in V1 (Ibrahim et al., 2016). Similarly, impairing the direct A1-V1 connectivity by cortico-cortical transections abolishes the sound-driven hyperpolarization of V1 (Iurilli et al., 2012). Compared to the described connectivity patterns between primary sensory cortices in rodents (Burkhalter, 1989; Wang and Burkhalter, 2007; Stehberg et al., 2014; Henschke et al., 2015), direct cortico-cortical projections are sparse in primate primary sensory areas, which has functional implications on cross-modal processing (Falchier et al., 2002; Clavagnier et al., 2004; Cappe and Barone, 2005). Single-cell recordings revealed only subthreshold neuronal responses in primate primary sensory areas (Molholm et al., 2002; Lakatos et al., 2007; Kayser et al., 2008), and suprathreshold multisensory neurons were restricted to higher cortical areas (Fu et al., 2003; Ghazanfar et al., 2005). In contrast to primate low-level areas where feedback cross-modal information only has a subthreshold influence on its postsynaptic targets (Allman et al., 2009), multisensory responses in rodent primary sensory cortices might rely on the direct cortico-cortical connections and less on feedback information from higher cortical association areas. This suggests that the presence or absence of multisensory suprathreshold effects might result from the number and strength of cross-modal inputs reaching rodent or primate primary sensory cortices respectively.

In contrast to the early cross-modal responses in primary sensory cortices, cross-modal effects occurring at longer poststimulus latency may be under the control of feedback information, which is sent via projection neurons from high- to low-level sensory areas (Smith et al., 2010; Banks et al., 2011). Recently, Morrill and Hasenstaub (2018) revealed that a minority of neurons in A1 responds at 40 ms after visual stimulus presentation, exceeding the time delay of monosynaptic information transmission. Inputs from higher sensory cortex, such as secondary visual cortex, might account for the occurrence of visual responses with a long latency in A1 (Bizley et al., 2007; Banks et al., 2011). Information between primary sensory cortices may also be transferred via a cortico-thalamic-cortical route (Hackett et al., 2007; Sherman, 2016). For example, Hackett et al. (2007) showed that thalamic nuclei (first-order medial geniculate complex and higher-order posterior nucleus of thalamus) share anatomical connections with somatosensory as well as with auditory cortex. This cortico-thalamo-cortical pathway might resemble the anatomical substrate of tactile information transfer from somatosensory to auditory cortex through first- as well as higher-order thalamus (Schroeder et al., 2001; Kayser et al., 2005).

Besides anatomical projections from higher sensory cortices, long-range prefrontal projection neurons have been proposed to modulate cross-modal responses in primary sensory cortices (Vaneden et al., 1992; Sellers et al., 2015; Zhang S.Y. et al., 2016). For example, Zhang S.Y. et al. (2016) identified retrogradely labeled neurons in the cingulate sulcus of PFC targeting V1. Furthermore, the anterior cingulate subdivision of PFC shares direct connections with V1, while primary and secondary motor cortices are connected to somatosensory and auditory cortex (Zhang S.Y. et al., 2016). The identified direct long-range projections between PFC and primary sensory cortices might act as anatomical substrate for the functional communication between low- and high-level areas during cross-modal processing. Future studies using virus-assisted circuit mapping and optogenetic manipulations shall unravel the contribution of top-down projections from PFC to primary sensory cortices during cross-modal processing.

### Mechanisms of Bottom-Up Cross-Modal Processing in Primary Sensory Cortices

Encoding of information requires coordinated neuronal firing that selectively filters relevant from irrelevant environmental information (Parker and Newsome, 1998; Connor et al., 2004; Harris and Mrsic-Flogel, 2013). Two neural communication codes – *rate coding* (i.e., changes in the frequency of action potentials) and *temporal coding* (i.e., changes of spike timing in relationship to the phase of network oscillations) – have been described (Oram et al., 2002; Kayser et al., 2009; Meredith and Allman, 2015). These two coding strategies often occur concurrently (Biederlack et al., 2006; Kayser et al., 2009; Bieler et al., 2017b), and as a result, increase the coding capacity (Tiesinga et al., 2008; Kayser et al., 2009; Figure 1Di). It is hypothesized that rate changes in single neurons code for the discrete properties of a stimulus, whereas temporal coding marks the relatedness of neuronal firing among neurons eventually leading to a coherent perception of the stimulus (Singer, 2009). Studies in the SC have identified two major operating principles of cross-modal processing. First, the more spatially and temporally coincident cross-modal cues appear, the greater is the multisensory *enhancement* (i.e., an increased neuronal response after cross-modal when compared to unimodal stimulation) (Meredith and Stein, 1983; Wallace et al., 1998). Second, the strength of the unimodal cues defines the magnitude of the cross-modal effect, such that weaker individual sensory stimuli evoke stronger cross-modal effects (*inverse effectiveness*) (Perrault et al., 2005). These principles of cross-modal integration served as a general guideline for deciphering cross-modal processing mechanisms in low-level sensory areas at single-cell and network level (Bieler et al., 2017b; Bieler et al., 2018).

Oscillatory activity reflects the rhythmic excitability fluctuations of neuronal populations within particular frequency bands that correspond to specific spatial scales of brain operation. This rhythmic nature of neural activity creates time windows during which inputs are more effective in driving the neurons. By making use of anatomical connectivity between and within brain networks, neuronal network oscillations account for local-global neuronal interactions as well as for maintaining persistent activity (e.g., during behavioral state) (Buzsaki and Draguhn, 2004; Buzsaki, 2010; Buzsaki and Wang, 2012). Synchronization of neuronal network oscillations subserves neuronal communication and enables the integration of sensory information across distant locations of the brain (Senkowski et al., 2008). Selective communication among neural networks might be achieved by coherence of oscillatory firing patterns (sending neurons) and gain modulation (receiving neurons) (Fries, 2015). Thus, rhythmic synchronization generates sequences of excitation and inhibition which focus the spike output of firing neurons and sensitivity to synaptic inputs of receiving neurons to a short temporal window.

Synchrony of activity in distant neural networks ultimately leads to the binding of anatomically segregated functional networks (Fries, 2005; Canolty et al., 2010; Canolty and Knight, 2010). Since unisensory networks encode relationships between detected information by synchronizing their activity, it raises the likelihood that similar mechanisms are involved in cross-modal processing. For example, information processing by one modality can enhance the population synchrony in lower-order regions responsive to another modality, such as primary sensory cortices or subcortical regions, in reciprocal relationship with other brain regions (Kayser and Logothetis, 2007; Driver and Noesselt, 2008; Tyll et al., 2011). This cross-modal synchrony enhancement of neuronal activity has been described for evoked as well as for induced responses: the impact of an external stimulus sensed by one modality is strengthened by appropriately timed information about the event in another modality (Figure 1Di; Sieben et al., 2013). Furthermore, the phase reset of spontaneous neuronal oscillations might facilitate the communication of distant neural networks during cross-modal processing (Figure 1Dii). The re-alignment of phases of ongoing neuronal oscillations in one processing region in relation to a cue of another sensory modality allows inputs to arrive at a high excitability phase (Lakatos et al., 2007; Kayser et al., 2008; Iurilli et al., 2012; Sieben et al., 2013; Figure 1Diii). In addition, the interaction of oscillations in different frequency bands, termed *cross-frequency coupling* (CFC), has been proposed as another mechanism of how distant brain regions synchronize their activity to interact (Canolty and Knight, 2010; Figure 1Div). The question arises whether CFC acts as a mechanism for the interaction of multiple sensory areas, and thus the integration of cross-modal inputs in rodent sensory cortices (Canolty et al., 2006; Schroeder and Lakatos, 2009). Recently, we examined the oscillatory interactions underlying CFC in a thalamo-cortical circuit during cross-modal processing (Bieler et al., 2018). Our study revealed a significant increase in beta-gamma phase-amplitude CFC between first-order thalamus and primary somatosensory cortex during cross-modal but not unimodal processing. Thus, the phase of the beta rhythm controls the power of coupled gamma oscillations through synchronization of the gamma amplitude envelope with the beta phase during cross-modal processing in thalamo-cortical networks.

While cross-modal effects at functional and anatomical level are widespread in primary sensory cortices, the exact configuration of a cross-modal stimulus ultimately defines which processing strategy, i.e., enhancement or depression of neural responses, is applied (Meijer et al., 2017).

### Mechanisms of Top-Down Modulation of Cross-Modal Processing in Primary Sensory Cortices

Several mechanisms of prefrontal top-down modulation of cross-modal processing in primary sensory cortices have been proposed (Tomita et al., 1999; Barceló et al., 2000; Monosov et al., 2011; Gilbert and Li, 2013; Teufel and Nanay, 2017). Temporal coding of neuronal excitability reflected by oscillatory activity in primary cortices might provide a temporal window for effective processing of top-down information (Figure 1Diii). Phase locking of oscillatory activity between PFC and primary sensory cortices was proposed to fulfill this role. In particular, oscillatory activity in primary sensory cortices creates temporal windows during which top-down PFC signals are more effective in driving neuronal activities in primary cortices during sensory processing. If this holds true, spikes from PFC arriving within temporal excitation windows of the sensory cortices might produce postsynaptic spikes in primary sensory cortices more effectively.

Several studies reported enhanced gamma synchronization between prefrontal and unisensory cortices during attention tasks. For example, Gregoriou et al. (2009) found a specific enhancement in gamma phase synchronization between frontal cortex and V4 during sustained attention in a covert spatial attention task (Gregoriou et al., 2009). Frontal locking of spikes to gamma activity in visual cortex encodes the attended location. Interestingly, frontal spike activity occurred ∼10 ms before the maximal excitability in visual cortex. This time delay might correspond to the transmission lag from frontal cortex to V4. Furthermore, the authors applied Granger causality analysis to study the directional coupling between PFC and V4. They showed that during the early stage of the task, when attention must to be shifted to a relevant location, frontal cortex initiated the oscillatory coupling across PFC and V4. Enhanced phase locking to gamma rhythm in V4 during the attention task was restricted to visual processing neurons, and did not include V4 neurons representing aspects such as visuo-movement or movement (Gregoriou et al., 2012). Of note, the gamma coherence between two distant brain regions may have an artifactual origin. It has been proposed that gamma coherence might reflect the coupling of two phase-locked network oscillations as well as the co-modulating effect of an upstream network common to both recorded networks (Buzsáki and Schomburg, 2015).

According to a largely accepted hypothesis, the PFC selectively facilitates the selection of task relevant information and enhances the representation of attended stimuli in primary sensory cortices (Baluch and Itti, 2011). To address this, Ardid et al. (2010) built a simulated model with weak coupling between two networks resembling a low-level sensory and a high-level brain area (Ardid et al., 2010). Enhanced gamma coupling between these two regions heavily influenced the synchronization between specific neurons encoding attended features across the areas. The results support the idea that inter-areal LFP coupling between PFC and primary sensory cortex selectively facilitates the communication between neurons encoding attention-related information. Several lines of evidence support the hypothesis that the top-down prefrontal signal effectively influences sensory processing in primary cortices. For instance, top-down attention affects V1 processing by enhancing the firing rate of neurons representing the attended stimulus (Treue and Trujillo, 1999; Bichot et al., 2005) and reducing the variability of inter-neuronal correlation (Cohen and Maunsell, 2009; Mitchell et al., 2009; Herrero et al., 2013). The reduced variability of correlation among neurons improves the signal-to-noise ratio for attention-relevant information and promotes efficient coding of attended features. Consequently, the signal-to-noise ratio improves (Cohen and Maunsell, 2009; Mitchell et al., 2009). Moreover, top-down attention modulates local oscillatory activity of primary sensory cortices in a frequency-specific manner (Gregoriou et al., 2015). For example, during attentional selection, neurons in visual and frontal areas encoding the attended location or feature synchronize their activity in gamma frequency (30–60 Hz) range (Tallon-Baudry et al., 2004; Bichot et al., 2005; Fries, 2005; Kreiter et al., 2005; Fries et al., 2008; Gregoriou et al., 2009). This might facilitate the propagation of information between these two areas (Salinas and Sejnowski, 2001; Azouz and Gray, 2003; Fries, 2005, 2009). In addition, reduced local alpha-beta oscillatory activity in V2 and V4 during an attention task (Thut et al., 2006; Fries et al., 2008; Siegel et al., 2008; Gregoriou et al., 2009; Buffalo et al., 2011) has been proposed to inhibit distracting inputs (Palva and Palva, 2007; Händel et al., 2011). Top-down attention also modulates the size and position of visual receptive fields, bursting activity, response latency as well as feature tuning of neurons (Murray and Wojciulik, 2004; David et al., 2008).

Investigation of local circuits and synaptic processes provide additional evidence for top-down modulation of cross-modal processing. Zhang et al. (2014) demonstrated that long-range glutamatergic projections from PFC modulate local circuits in V1 (Zhang et al., 2014). Optogenetic activation of prefrontal neurons led to enhanced responses of V1 neurons. Light stimulation of prefrontal axonal terminals in V1 induced center-surround modulation, which increased the response at the activation site, while suppressing the response at a nearby location. Three subtypes of interneurons in local visual circuits were targeted by top-down prefrontal modulation. First, somatostatin-positive interneurons (SOM^+^) were critical for surround suppression, since they inhibited the response of pyramidal neurons to the prefrontal input within a 200 μm radius. Second, vasoactive intestinal peptide-positive interneurons (VIP^+^) were crucial for center facilitation in V1 (Fu et al., 2014), mediating the disinhibition of pyramidal neurons. This disinhibition effect was mainly localized at the site of prefrontal axons in V1 and caused the increase of attention-inducing firing rate. Third, parvalbumin-positive (PV^+^) GABAergic interneurons were required for long distance inhibition, since their inactivation reduced prefrontal axon-induced inhibitory inputs at a distance of 400 μm. Thus, long-range prefrontal projections act through local microcircuits to exert top-down modulation of sensory processing.

## The Emergence of Sensory-Cognitive Interplay During Cross-Modal Development

The brain’s ability to create a coherent perception of the environment by integrating information of various sensory modalities is not present immediately following birth. The development of cross-modal integrative capabilities is a protracted process both in rodents (Ghoshal et al., 2011; Mowery et al., 2016; Hattori and Hensch, 2017) as well as in humans (Scheier et al., 2003; Lewkowicz and Ghazanfar, 2009; Lewkowicz, 2010). This process depends on the alteration and refinement of neural circuitry following uni- and cross-modal sensory experiences.

Cross-modal abilities mature under the influence of intrinsic (i.e., genetic cues) and extrinsic (i.e., environment) factors (Rauschecker et al., 1992; Yu et al., 2010; Frangeul et al., 2016; Moreno-Juan et al., 2017). During embryonic development, molecular cues and genetic programs control the generation, migration, and differentiation of neurons as well as the formation of rudimentary connectivity (Toda et al., 2013; Diao et al., 2018; Telley et al., 2019). At later stages, but before the onset of sensory transduction, spontaneous electrical activity occurring in distinct spatial and temporal patterns refine rudimentary connectivity and facilitate the formation of sensory maps (Galli and Maffei, 1988; Dehorter et al., 2012; Luhmann et al., 2016; Anton-Bolanos et al., 2019). The patterns of spontaneous network activity are conserved across species, and their perturbation causes deficits in network refinement (Huberman et al., 2008). During defined developmental periods (i.e., critical/sensitive periods) the circuits, and later behavioral abilities, are particularly prone to being shaped by experience-dependent electrical activity (Chapman, 2000; Chang and Merzenich, 2003; Pfeiffenberger et al., 2006; Ghoshal et al., 2009; Khazipov et al., 2013). The patterns of electrical activity are similar in age-matched rodents and humans (Khazipov and Luhmann, 2006).

### Development of the Tactile System

**FIGURE 2:**
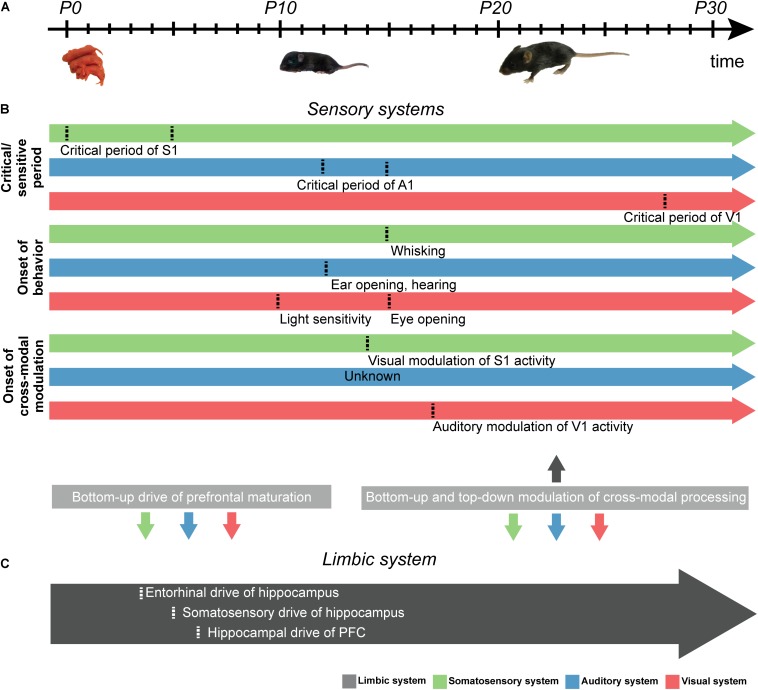
Schematic diagram displaying the developmental milestones of sensory and limbic development in rodents. **(A)** Schematic illustration displaying the developmental timeline of sensory development from postnatal day (P) zero onward. **(B)** Schematic arrows showing the time points (marked by dotted line) of (i) the critical/sensitive period of somatosensory (green), auditory (blue), and visual (red) development, (ii) the onset of unisensory behavior, and (iii) the start of cross-modal modulation. Uni- and cross-modal inputs in the first days of life are hypothesized to drive the development of the limbic system in a bottom-up manner, while bottom-up as well as top-down interactions between the primary sensory cortices and limbic system are present at later stages of development (gray boxes, bottom). **(C)** Same as **(B)** for PFC. Time points shown in gray arrow mark developmental milestones of limbic system development.

By using their highly sensitive whiskers, nocturnal rodents can acquire tactile information and build spatial representations of the environment (Petersen, 2007). Whisker-related inputs are processed in somatotopic maps where each whisker is represented by a discrete anatomical unit (“barrel”). Barrel-like cell aggregates form soon after birth (Jhaveri et al., 1991; Schlaggar and O’Leary, 1994). Early sensory experience is mandatory for the development of somatosensory processing. Neonatal whisker trimming from birth on impairs the dendritic complexity of neurons in the barrel cortex and behavioral performance in the gap-crossing task during adulthood (Carvell and Simons, 1996; Lee et al., 2009). Whisker-dependent exploratory behavior does not develop until the second postnatal week (Welker, 1964; Figures 2A,B). This suggests that prior to experience-dependent plasticity other mechanisms must contribute to the development of somatosensory perception. Transcription factors, such as Gbx2, Mash1, and Pax6 have been reported to be involved in pathfinding of axons from thalamus to S1 (Tuttle et al., 1999; Hevner et al., 2002). In addition, discontinuous electrical activity, which appears within the first two postnatal weeks, shapes the development of topographic organization in S1. Several patterns of neonatal electrical activity have been characterized, such as gamma oscillations, spindle bursts with frequencies in theta-beta range, and long-oscillations (Yang et al., 2009; Minlebaev et al., 2011; Yang et al., 2016). Peripheral inputs are not mandatory for the emergence of these early activity patterns. Gamma oscillations and spindle bursts remain after the peripheral pathways were lesioned (Khazipov et al., 2004; Minlebaev et al., 2011; Yang et al., 2013). Early activity patterns may act as a template for the emergence of cortical topography. For instance, the volume of synchronized neurons during spindle burst activity reflects the anatomical size of the future barrels (Yang et al., 2016). Long oscillations are assumed to synchronize large neuronal networks and boost the formation of functional neuronal ensembles (Yang et al., 2009). With ongoing maturation, rodents start to whisker and early tactile experience further refines the somatosensory circuits.

### Development of the Auditory System

Similar to tactile development, the maturation of auditory pathways containing orderly representations of frequency selectivity involves both molecular cues and spontaneous electrical activity. For example, neurotrophins such as BDNF and NT-3, ephrins (Ernfors et al., 1992; Hossain et al., 2008) and semaphorins (Gu et al., 2003; Webber and Raz, 2006) have been reported to guide auditory innervation. Spontaneous electrical activity further refines and maintains the tonotopic architecture set by molecular cues (Wang and Bergles, 2015). In rodents, the ability to respond to acoustic stimuli emerges around postnatal (P) day 12 (Uziel et al., 1981; Kelly, 1992; Figures 2A,B). Experience-dependent activity then promotes the fine-tuning of auditory networks (Friauf and Lohmann, 1999). Before this age, environmental factors regulate the maturation of auditory processes. For example, early interactions with the mother modulate the maturation of the auditory system in pups (Cárdenas et al., 2015). Auditory reflexes in pups were accelerated when the mothers were reared in an enriched environment during gestation. Moreover, exposure to frequency-enriched acoustic environments during the first 14 days after birth significantly decreased the threshold of auditory responses in a frequency-specific manner (Chang et al., 2018). Rearing in a disturbed acoustic environment impairs the development of the auditory system (Zhang et al., 2002; Chang and Merzenich, 2003; Nakahara et al., 2004; Speechley et al., 2007). Early noise exposure induced permanent structural changes in the rat auditory system (Ouda et al., 2016). Rat pups exposed to trains of 5 kHz pure tones showed larger regions of auditory cortex tuned to 5 kHz at adulthood (Han et al., 2007). Thus, over-representations of certain frequencies during early development likely reduces auditory discrimination.

### Development of the Visual System

Rodents are born blind. The retina starts to be light-sensitive during the second postnatal week, and shortly after that, the eyelids open (Figures 2A,B; Sernagor, 2005). From birth on, axonal projections from the lateral geniculate nucleus (LGN) target cells in the granular layers of V1 leading to the initiation of cortical topographical organization. During early development, when the retina is light-insensitive, bursts of action potentials (i.e., retinal waves) emerge under the control of the cholinergic system (Brombas et al., 2017) and propagate across the retina (Wong et al., 1993). These retinal waves are transmitted via the optic nerve to the LGN and finally to V1, where they boost cortical spindle bursts (Hanganu et al., 2006). At each developmental stage of V1, retinal waves differ in their properties, thereby instructing the development of visual feature processing mechanisms (Huberman et al., 2008).

With the onset of light sensitivity, visual experience shapes the cortical topography (Smith and Trachtenberg, 2007). In cats, monocular visual deprivation led to a size reduction of columns corresponding to the sutured eye, whereas columns corresponding to the non-deprived eye expanded (Hubel et al., 1977; Le Vay et al., 1980). Visual deprivation during the sensitive period leads to alterations in thalamo-cortical connectivity (Fox and Wong, 2005; Hofer et al., 2008) and as a consequence alters the input organization from both eyes (Espinosa and Stryker, 2012). Experience has been shown to control the tuning of V1 neurons to stimulus orientation and direction (Hubel and Wiesel, 1962; Weliky et al., 1996). Thus, even though coarse orientation selectivity emerges under the influence of experience-independent neuronal activity (White et al., 2001), high-level orientation selectivity appears only in the presence of visual inputs (Chapman and Stryker, 1993). In contrast, neither molecular cues nor spontaneous activity, but visual experience seems to be required for tuning V1 neurons to stimulus direction (Li et al., 2006).

### External Inputs Controlling the Development of Cross-Modal Processing in Rodent Primary Sensory Cortex

While a wealth of studies documented the relevance of early electrical activity for the maturation of topographic organization, few studies addressed the mechanisms of cross-modal development in primary sensory cortices. One key question is whether perturbing unisensory development – even prior to full responsiveness of all stimulus-related sensory modalities – has long-lasting consequences for the development of cross-modal processing. It appears that cross-modal development requires a certain level of unisensory maturity (Ghoshal et al., 2011; Sieben et al., 2015). For instance, Sieben et al. (2015) showed that tactile deprivation shortly after birth (P0-5) causes abnormal visual-tactile cross-modal processing later in life. Furthermore, it has been shown that the power and phase of neuronal activity were modulated by cross-modal stimuli of juvenile rats with only minimal cross-modal experience (i.e., closed eye lids, but light-sensitive retina and tactile sensation in P14-16 rats) (Bieler et al., 2017a). Thus, network interactions ensuring cross-modal processing emerge before cross-modal experience and refine during juvenile development (Figure 2B).

### Development of the PFC

As previously mentioned, the PFC is involved in memory, attention, and decision-making (Miller, 2000; Vertes, 2006). In addition, it is considered to act as a hub of cross-modal processing (Fuster et al., 2000; Nieder, 2017). Overall, the PFC follows the developmental milestones described for primary sensory cortices. Early patterns of oscillatory activity are highly discontinuous and temporally fragmented (Brockmann et al., 2011), yet they emerge a few days later when compared with V1 or S1. Moreover, the maturation of the PFC is remarkably prolonged when compared to other cortical areas (Leipsic, 1901; van Eden and Uylings, 1985). The prefrontal cytoarchitecture and correspondingly, the executive and mnemonic abilities, are not fully developed until adolescence (van Eden and Uylings, 1985).

The functional development of PFC seems to be controlled by activity in the intermediate/ventral hippocampus (HP). Hippocampal theta bursts emerging a few days before prefrontal spindle bursts, drive the generation of neonatal prefrontal oscillations by phase-locking the neuronal firing via axonal pathways (Brockmann et al., 2011). Remarkably, the early entrainment of prefrontal-hippocampal networks is critical for the mnemonic ontogeny at juvenile stage (Krüger et al., 2012). During later development (∼P10), the oscillatory activity in both PFC and hippocampus switches from discontinuous bursts to continuous theta-gamma oscillations. This switch occurs almost simultaneously in the prefrontal and primary sensory cortices (Colonnese and Khazipov, 2010).

### Sensory-Cognitive Interactions During Development

As outlined in sections “Development of the Tactile System,” “Development of the Auditory System,” and “Development of the Visual System,” early endogenous and sensory-driven activity patterns contribute to the development and refinement of neuronal networks (Hanganu et al., 2006; Minlebaev et al., 2009; Yang et al., 2009; Yang et al., 2013). Perturbing sensory inputs during critical/sensitive periods of development has profound effects on the neuronal activity and its underlying anatomical connectivity, and thus affects behavior (Fagiolini et al., 1994; Carvell and Simons, 1996; Erzurumlu and Gaspar, 2012; Levelt and Hubener, 2012; Kral, 2013).

Perturbation of a sensory input leads to anatomical and functional modifications in the remaining sensory systems. As a consequence, neurons adaptively reorganize to integrate the function of other sensory systems, in a process termed *cross-modal plasticity* (Bavelier and Neville, 2002; Lee and Whitt, 2015). Cross-modal plasticity alters perceptual abilities. For example, several studies have shown that bilateral lid suture or enucleation impairs orientation and direction selectivity of V1 neurons, but enhances the processing of auditory and somatosensory inputs in V1 (Rauschecker et al., 1992; Rauschecker and Kniepert, 1994; Yaka et al., 2000; Izraeli et al., 2002). Similar cross-modal activation patterns after sensory deprivation have been observed in other primary sensory cortices (Goel et al., 2006; Hunt et al., 2006; Lee and Whitt, 2015; Meng et al., 2015).

Recently, the effects of non-visual inputs on experience-dependent plasticity in V1 during early postnatal development have been investigated (Hattori and Hensch, 2017; Figure 2B). Concurrent visual-auditory inputs impaired the development of orientation selectivity of V1 neurons if they occurred before or after the critical period. However, the effect was dampened if cross-modal visual-auditory stimuli occurred during the critical period. The authors suggest that this effect is likely caused by a sound-driven balance of suppression and enhancement of V1 spiking activity, which is required for the tuning and consolidation of visual selectivity. Similarly, it has been shown that the onset of visual experience controls the development of auditory processing (Mowery et al., 2016). In particular, the critical period of auditory development was precociously closed by early eyelid opening and extended by delayed eyelid opening (Figure 2B).

Few experimental data have documented the impact of altering the functional anatomy and neuronal activity of primary sensory cortices on the development of PFC (Kolb and Gibb, 2015). It has been shown that sensory deprivation increases the density of interneurons in PFC (Ueno et al., 2015). This is in line with findings from primary visual cortex where the laminar distribution of PV^+^ neurons is altered following enucleation (Desgent et al., 2010). Overall, a mechanistic understanding of the effects of sensory deprivation on the bidirectional communication between primary sensory cortices and PFC is currently lacking.

As discussed in section “External Inputs Controlling the Development of Cross-Modal Processing in Rodent Primary Sensory Cortex,” perturbations of unisensory development prior to full maturation of all unisensory systems has long-lasting consequences for the development of cross-modal processing abilities (Ghoshal et al., 2011; Sieben et al., 2015). Notably, during the sensitive period of tactile development, the functional maturation of the PFC is boosted by the excitatory drive from the hippocampus (Brockmann et al., 2011; Bitzenhofer et al., 2017; Ahlbeck et al., 2018; Figure 2C). However, it is largely unknown how early sensory development affects the maturation of the limbic system. Several studies have shown that sensory experience is important for synaptic pruning during PFC development (Schanberg and Field, 1987; Richards et al., 2012). For example, raising rodents in a tactile-enriched environment from birth on increases the prefrontal spine density and improves the performance in PFC-dependent tasks at adulthood. The increased dendritic branching and spine density in PFC (Kolb et al., 2012; Kolb and Gibb, 2015) argue for significant plastic changes occurring when experiencing a sensory enriched environment. Thus, sensory-driven activity might directly impact the maturation of the limbic system.

Early electrical activity in sensory and limbic circuits may facilitate the network development required for their communication (Mohns and Blumberg, 2008). Neocortical spindle bursts are induced by proprioceptive feedback which is initiated by twitches of the distal limbs (Khazipov et al., 2004). These spindle bursts drive the activation of CA1 neurons and critically depend on neocortical-hippocampal interactions (Mohns and Blumberg, 2010). Since myoclonic movements induce bursts of activity in the medial entorhinal cortex, which in turn drives hippocampal responses, it has been suggested that entorhinal-hippocampal interactions are part of a large-scale bottom-up circuit activated during neonatal movements (Valeeva et al., 2019). While the impact of somatosensory processing on limbic system development began to be elucidated, it is currently unknown whether similar bottom-up interactions exist for other sensory systems. Similarly, the impact of top-down PFC activity on early sensory development and its importance for adult cross-modal processing capabilities are still unknown.

## Animal and Human Research as Background for Brain-Inspired Intelligent Robotics

Neuroscientific insights can be harnessed to build adaptive and intelligent machines. Given recent advances in calcium (Ca^2+^) imaging using genetically encoded Ca^2+^ indicators and in the use of optogenetic tools for causal manipulation of neural circuits (Fenno et al., 2011; Grienberger and Konnerth, 2012), current and future research can provide a plethora of insights into the neuronal computations of cross-modal processing. Based on brain-like neural architectures and biologically plausible learning mechanisms (Pitti et al., 2009), computer implementations can create robot perception and action (Floreano et al., 2014). The field of robotics is one of the most dynamic areas of technological development (Zhang B. et al., 2016), and robots performing very specific tasks are increasingly found in industry, service, and medicine. A growing field is also the interplay between robotics and neuroscience. For instance, equipping cognitive robots with the ability to process and integrate cross-modal information streams ensures that they will interact with the environment more efficiently, even under conditions of sensory uncertainty (Parisi et al., 2019). Similarly, developmental robotics, which is motivated by human cognitive and behavioral development, aims to provide a better understanding of the development of cognitive processes using robots with rich sensory and motor capabilities as testing platforms (Breazeal and Scassellati, 2002; Lungarella et al., 2003; Prince, 2008; Cangelosi and Schlesinger, 2015, 2018).

As outlined above, low-level sensory and high-level neural networks accounting for cognitive processing interact in a bottom-up and top-down manner to create a coherent perception of the multisensory environment. Similarly, bottom-up and top-down processing underlying the integration of multipleisensory information streams play a crucial role in the development of autonomous agents and cognitive robots. However, these two research streams often developed independently. Closer interactions between them appear mutually beneficial for several reasons. First, biological inspiration for the modeling of bottom-up cross-modal processing in robots is of crucial interest in order to endow agents with improved robustness, flexibility and performance, particularly in the case of uncertain, ambiguous or incongruent cross-modal inputs (Parisi et al., 2019). For example, biological inspiration has played a major role in the field of odor-guided navigation (Russell, 2001). Bailey et al. (2005) developed a robot with multisensory processing capabilities, and in particular stellar odor-tracking performance similar to that found in animals, in order to locate the source of chemical plumes (Bailey et al., 2005). Barsky et al. (2019) applied a deep learning method to combine disparate sensory inputs, such as auditory and visual information. Cross-modal processing facilitated the learning of a humanoid drumming robot to generate suitable motion sequences to match desired unseen audio or video sequences (Barsky et al., 2019). Axenie et al. (2016) proposed a novel audio-visual sensory processing architecture for robust multisensory fusion in robotic systems, which is inspired by the distributed macro-architecture of the mammalian cortex (Axenie et al., 2016).

Second, biological inspiration for the modeling of top-down cross-modal processing in robots is mandatory for autonomous agents and cognitive robots to develop perception through active groping. Fujimoto et al. (2009) developed a robot being able to pick up dishes based on active groping. The robot roughly formulated a strategy for selecting dishes placed close to each other. Subsequently, by actively acquiring the geometric information of the dishes during the implementation of the strategy, the robot was able to efficiently complete the task (Fujimoto et al., 2009). Inoue (1971) developed a robot to search for a block by actively moving the hand along a predefined track and detecting contact with items using touch sensors (Inoue, 1971). Maekawa et al. (1992) developed a finger-shaped tactile sensor which could reconstruct the shape of an object by actively moving along a predefined grid and detecting the position and direction of contact by using sensors (Maekawa et al., 1992). These studies demonstrate that robots have the capability to progressively learn in an ever-changing multisensory environment by means of self-exploration and social interaction.

However, robots are still limited in their dynamic movements, emotional perception and adaptive interactions with humans, and this drawback limits their application (Wiese et al., 2017; Cross et al., 2019). To overcome this challenge, brain-inspired intelligent robotics may equip systems with advanced human-like cognitive abilities such as improved multisensory processing and learning capabilities by mimicking the structures and mechanisms underlying sensory-cognitive processing (section “Sensory-Cognitive Interplay During Cross-Modal Processing”). In fact, multisensory perception has been named as one of the key sensory-cognitive functions in order for cognitive robots to thrive in a complex and dynamic environment (Zhang B. et al., 2016). A lack of multisensory perceptive capabilities, makes it more sophisticated to acquire other cognitive computations and to function autonomously. Continuous learning of robotic systems is crucial, because internal models of the multisensory world must be acquired and adapted throughout development in order for multisensory processing capabilities to emerge (section “The Emergence of Sensory-Cognitive Interplay During Cross-Modal Development”) (Rohlf et al., 2017). Recent endeavors led to the creation of an open source humanoid called NICO (Neuro- Inspired COmpanion), which due to its flexible design and open and modular hardware and software framework can adapt to individual experimental set-ups and opens the door to multimodal human-robot interaction research with the aim of developing autonomous agents and cognitive robots (Kerzel et al., 2017).

## Conclusion and Future Lines of Research

It has been hypothesized that the bottom-up sensory drive contributes to establishing neuronal circuits in the limbic system during early development (Mohns and Blumberg, 2008). At adulthood, the interaction between low-level sensory and high-level limbic areas enables cross-modal perceptual decision-making. Cross-modal representations are transferred from primary sensory cortices to PFC in a bottom-up manner, and the representation of an attended stimulus in primary sensory cortices is selectively enhanced by top-down prefrontal modulation (Bizley et al., 2016). However, the interactions between primary sensory cortices and PFC during bottom-up/top-down cross-modal processing have been poorly characterized. To this end, techniques that specifically manipulate neuronal pathways between PFC and primary sensory cortices are necessary. Relying on recent advances in optogenetic terminal field excitation/inhibition, selectively illuminating axon terminals originating from PFC and targeting primary sensory cortices, would allow for the manipulation of the direct pathways between PFC and primary sensory cortices. This pathway-specific targeting will link function and connectivity underlying cross-modal processing within sensory-limbic circuits.

## Author Contributions

XX, IH-O, and MB contributed to the conception and design of the study, and wrote sections of the manuscript. XX and MB organized the literature data base and wrote the first draft of the manuscript. All authors contributed to manuscript revision, read and approved the submitted version.

## Conflict of Interest

The authors declare that the research was conducted in the absence of any commercial or financial relationships that could be construed as a potential conflict of interest.
